# Level of health literacy and associated factors among Jimma town public high school adolescent students: A cross-sectional study

**DOI:** 10.1371/journal.pone.0315365

**Published:** 2024-12-16

**Authors:** Merga Garoma Jatu, Desalew Tilahun Beyene, Dechasa Befikadu W. Senbat, Tesfaye Asfaw Alemayehu, Diribsa Tizazu Hailu, Serkalem Aschalew Jima, Midhagsaa Dhinsa Kitila, Ebissa Bayana Kebede

**Affiliations:** 1 Department of Nursing, Institute of Health Sciences, Dambi Dollo University, Dambi Dollo, Ethiopia; 2 School of Nursing, Faculty of Health Sciences, Institute of Health, Jimma University, Jimma, Ethiopia; 3 Department of Medical Laboratory Science, Institute of Health Sciences, Dambi Dollo University, Dambi Dollo, Ethiopia; 4 School of Nursing and Midwifery, College of Health and Medical Science, Haramaya University, Harar, Ethiopia; 5 Department of Nursing, College of Medicine and Health Science, Arsi University, Assela, Ethiopia; 6 Department of Nursing, College of Health and Medical Sciences, Dilla University, Dilla, Ethiopia; North-West University, SOUTH AFRICA

## Abstract

**Objectives:**

This study aimed to assess the level of health literacy and its associated factors among Jimma town public high school adolescents, Jimma, Oromia, Southwest Ethiopia, 2023.

**Methods:**

A facility-based cross-sectional study was conducted in June 2023 among 604 Jimma town public high school adolescents. A multistage sampling technique was employed to recruit study participants. A pretested self-administered questionnaire was used to collect data. Data was entered into epidata 4.6 and exported to SPSS version 26.0 for analysis. Bivariable and multivariable logistic regressions were performed to identify factors associated with the level of health literacy. P-values less than 0.05 at 95% CI were used to declare statistically significant associations. The results were presented by text, tables and charts as necessary.

**Results:**

From 634 total sample size about 604 participated. About 317 (52.5%) were males. The age of participants ranged from 14 to 19 years, with a mean age of 16.95+1.52. The finding revealed that only 35.26% (95% CI; 31.44, 39.09) of participants had desired health literacy, while 64.74% of them had limited health literacy levels. Age of 18–19 [AOR = 3.99(2.41, 6.60): p<0.001], being in 11–12 grade level [AOR = 2.38(1.44, 3.95); p = 0.001], being from currently employed father [AOR = 4.20(1.98, 8.92); p<0.001] and being from currently employed mother [AOR = 4.54(2.82, 7.31); p<0.001] were factors positively associated with the level of desired health literacy.

**Conclusion:**

Since a significant number of students in our study area had limited health literacy, we recommend schools to integrate a school health service that contains health education services. Moreover, efforts should be undertaken to raise adolescent health literacy for middle adolescents, early adolescents, and students whose families were not employed.

## Introduction

Health literacy (HL) is a relatively new concept that has grown significantly in its relevance to public health [1, 2]. The word "health literacy" (HL) was initially used by Simonds in relation to health education in schools in 1974 [3]. It is now described as a person’s capacity to get, understand, evaluate, and use of information, and services for their well-being [4]. Health literacy can be functional health literacy that involves basic reading and writing skills, interactive health literacy which contains everyday activities and communication, and critical health literacy critically evaluates information to influence life events [5]. Health Literacy (HL) is a useful tool for empowering adolescents to manage their well being [6]. People with limited levels of health literacy are more likely to have risk of mortality and morbidity [7].

Limited health literacy is a major global problem of many adolescents [8]. Evidences indicate that nearly half of adolescents have limited health literacy [9]. The pooled prevalence of limited HL ranged from 27% to 48%, making it a public health concern across Europe [10]. Limited health literacy is also a serious problem in many low-and-middle-income countries (LMICs) because of low levels of education and inadequate health services [11].

Despite the scant data and significant variation among studies, Southeast Asian countries have 55.3% of limited health literacy [12]. Although health literacy levels in Africa tend to differ across countries, limited HL is widespread among adolescents [13]. Findings from Sub-Saharan African countries also revealed that 57.87% of adolescents had limited health literacy [14]. Earlier studies on adults in clinical settings in Ethiopia found that 40.9% and 43.9% of participants had limited health literacy levels, respectively [15, 16].

People with inadequate health literacy do not perceive factors such as smoking, diet, obesity, alcohol, physical activity to be important disease risk [17]. Around 8% of adolescents suffer from chronic illnesses, which are further worsened by limited health literacy [18]. HL is affected by education level, gender, parental education, external support, economic situation, expenditure on mobile phones, and home book collection [19].

The World Health Organisation developed the Ophelia strategy approach to optimise HL and access to identify community health literacy issues and create solutions for them [20]. In the USA, the National Action Plan to Improve Health Literacy (NAPIHL) encourages diverse sectors to play a role in improving health literacy; however, most programs are focused on adults who are actively participating in the health care system [21].

Adolescents’ health literacy improves if intervened at the community and school levels [22]. A comprehensive framework is needed to address limited health literacy, shifting focus from individual educational interventions to supporting actions at higher levels of influence [23]. Previous studies have shown that health literacy strategies can dramatically increase health promotion behaviours and health literacy status [24].

Providing essential information and skills can enhance health literacy, enabling individuals to maintain and improve their own health, as well as those of their family and environment [25]. Despite global health literacy policies and programs, evidence and intervention tools for community practitioners are not being developed promptly [26].

Despite the effectiveness of interventions like materials and personalized educational sessions in enhancing health literacy, they are frequently time and resource-intensive [27]. The existing literatures indicate the need for further appropriate health literacy assessment and effective interventions in adolescents [28]. Despite its growing importance, adolescents in developing countries are not receiving the same level of attention as adults due to a lack of HL research in schools [29–31].

Studies on health literacy in Ethiopia have been limited, focusing mainly on clinical settings and adult populations. This study aimed to assess the health literacy and its associated factors among the adolescents of Jimma town public high school. This study aids Jimma town high school adolescents in improving their health literacy, enabling families, teachers, and health departments to plan and intervene in existing gaps.

The findings of this study will serve as baseline data for improving adolescent health literacy in Jimma town high school, promoting health promotion, and providing input for future research.

## Methods and materials

### Study setting

This study was conducted in Jimma town, which is the capital town of Jimma zone, located in the Oromia region, 352 km South-west of Addis Ababa. Based on the 2007 census conducted by CSA, Jimma town has a total population of 120,960, of which 60,824 were male and 60,136 were female. Age distribution shows that about 28.7% were below the age group of 14, whereas those who were in the working age group (15–64) and old age (>64) were 69.5% and 2.8%, respectively [32].

Jimma town is divided into 17 kebeles. The town has two public hospitals, four health centers, 22 health posts, 54 primary schools and 15 high schools. The town has eight public and seven private high schools (grades 9–12) with 14,681 students registered for the academic year of 2015 E.C. More than three-fourths of the students (12,635) were from public high schools and the remaining 2,046 were from private high schools. The study was conducted in June 2023.

### Study design

Facility-based cross sectional study was conducted.

### Populations

All adolescents aged 10–19 who were attending public secondary school education in Jimma town were considered the source population. The study population consisted of all the randomly selected adolescent secondary school students that were aged 10–19 years.

### Sample size determination and sampling techniques

The sample size for the first objective (level of health literacy among Jimma town high school students) was determined using the single population proportion formula with the following assumptions: Z = the standard normal deviation at the 95% confidence level; = 1.96; d = the margin of error that can be tolerated; 5% (0.05); and design effect = 1.5. Sample size was calculated based on the assumption that 50% of participants had adequate health literacy levels.


$$\text{n}=\frac{\left(\text{Z}\frac{\alpha }{2\;}\right)2\;\text{p}\left(1-\text{p}\right)}{\text{d}\;2}(\text{deff})=\frac{\left(1.96\right)2\;0.5\left(1-0.5\right)}{\left(0.05\right)2}(1.5)=576$$

After adding 10% non-response rate, the final sample size became 634.

In Jimma, there were 8 public high schools, comprising a total of 12,635 students. Three of these eight high schools were randomly selected using lottery method. The selected three schools comprised more than half (7358) of the total students in Jimma town public high schools. Of these students, 6929 were in the adolescent age group. A multistage sampling technique using stratified sampling and simple random sampling techniques was used to select the study participants.

**Fig 1:**
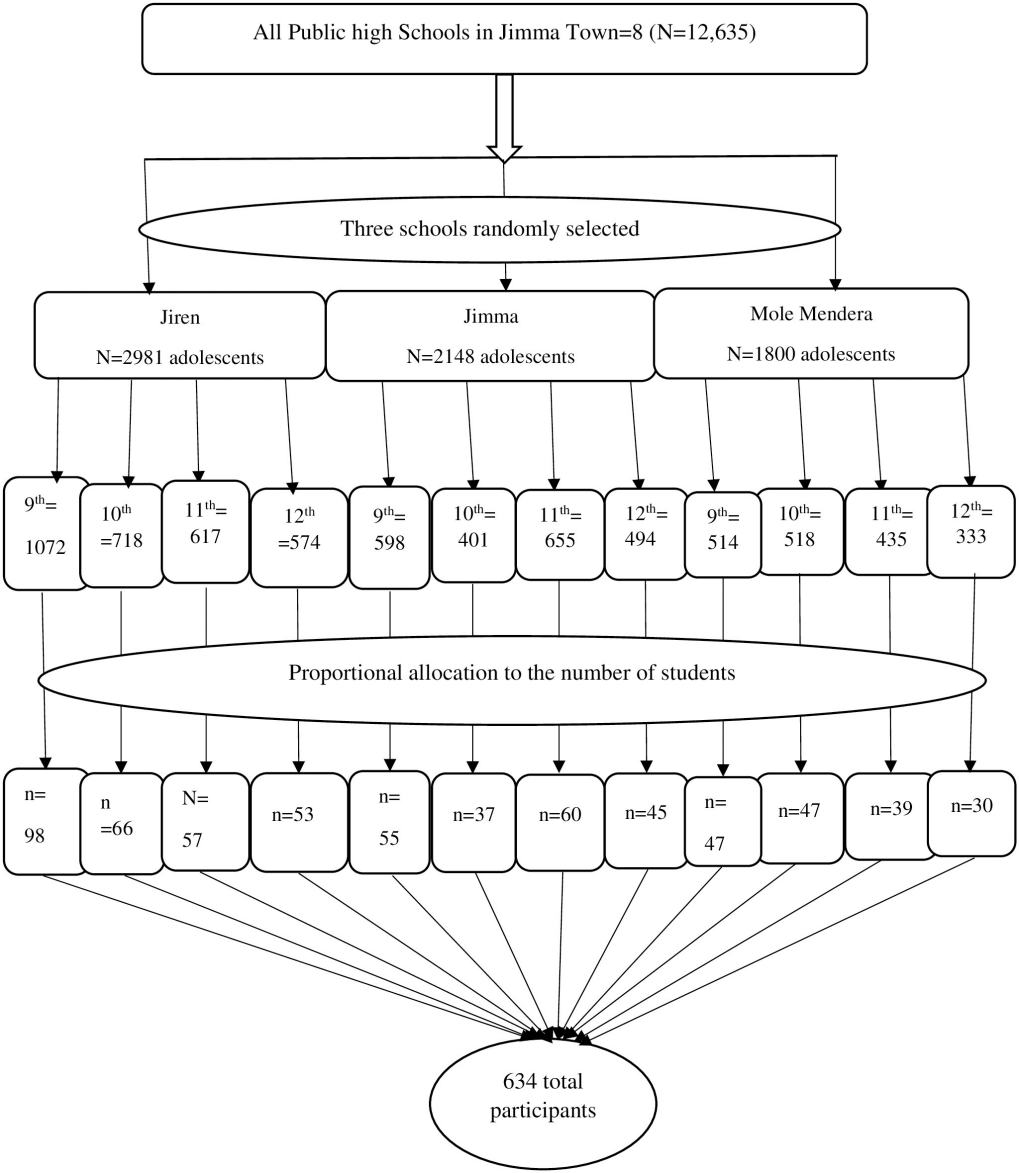
Schematic representation used in the selection of Jimma town public high school adolescent students in Jimma town, Southwest Ethiopia (n = 604).

Initially, three of the eight high schools were randomly selected using lottery method. In the following phase, students from randomly chosen schools were divided into grades ranging from 9 to 12 grades. Following the proportional allocation of study participants based on class size, the participant students were selected using a simple random sampling technique from the list of students in each grade at each school using a computer-generated random number. A self-administered questionnaire was provided for the selected students during the break of their class (Fig 1).


$$n=\;\frac{number\;of\;adolescent\;students\;in\;each\;grade*total\;sample\;size}{total\;number\;of\;schools\;adolescent\;students}$$

### Study variables

#### Dependent variables

Level of health literacy.

#### Independent variables

*Sociodemographic factors*. Age, gender, grade level, marital status, area of residence, number of family member, father´s education level, mother’s education level, father’s job and mother´s job.

*Sources of health information*. Teachers, parents, health professionals, internet, magazines, books, radio and television.

*Social support*. Social support from family, social support by friends and significant others.

*School related factors*. Academic performance, health related topic education, frequency of health related topic education, interest in health topic education, participation in school clubs, school attention on health promotion and school environment.

### Operational definitions

#### Health literacy

Ability of individuals and communities to access, understand, appraise and use information and services to make decisions regarding health [20].

#### Desired health literacy

A literacy status marked for a respondent with score of >66% as measured by health literacy measure of adolescents [33].

#### Limited health literacy

A literacy status marked for a respondent with score of 0–66% as measured by health literacy measure for adolescents [33].

#### Adolescents

Individuals aged 10 to 19 years [34].

#### Academic performance

Self-perceived school performance compared to peers as filled by students.

#### Multidimensional perceived social support scale (MPSS)

Is a measurement of perceived social support (emotional, instrumental, informational, and appraisal) from family, friends, and significant others [35].

#### Low MPSS

Mean value of 0–2.9 when measured with MPSS [36].

#### Medium MPSS

Mean value of between 3–5 as measured with MPSS [36].

#### High MPSS

Mean value >5 as measured using MPSS [36].

#### School environment scale

Instrument used to measure students’ subjective feelings about opportunities and rewards for prosocial involvement in schools [37].

### Data collection procedure and measurements

Data were collected using an adapted structured self-administered questionnaire. A validated instrument was used for determining the level of health literacy, which was adapted from Health Literacy Measure for adolescents developed by Ghanbari [33]. The tool was prepared in English. The tool is customised and contextualised to suit the specific population or setting being studied. Before being administered to adolescents, the questionnaire is submitted to seven experts for consultation and modified according to their comments.

Based on the pre-test results, the necessary revisions were made to increase the messages’ simplicity, clarity, and understandability. The questionnaire was translated into local languages such as Amharic and Afan Oromo and re-translated to English to maintain consistency in translation. The questionnaire consisted of five parts with a total of 81 items.

The first part included 12 questions on the socio-demographic characteristics of the students. The second part consisted of 1 multiple response item on the sources of health information. The third part included seven items school-related factors and 9 items on school environment scale measurements [36, 38–51].

The School Environment Scale which was used to assess the school environment was adapted from the Communities That Care Youth Survey [37]. It measures students’ subjective feelings about opportunities and rewards for prosocial involvement, with respondents rating each statement on a 4-point Likert scale (1 = strongly disagree to 4 = strongly agree).

The SES total score ranges from 9 to 36, with higher scores suggesting stronger bonds of attachment to school [48]. The reliability and validity test performed by Guo et al. showed that SES had high internal consistency (Cronbach’s α = 0.88) and appropriate construct validity. SES had strong internal consistency in this study (Cronbach’s α = 0.80).

The fourth part had 12 items on a multidimensional scale of perceived social support from three sources of individuals’ social lives: family, friends, and significant others. It makes use of a 7-point Likert-type scale for its measurements, with ratings from "1 = very strongly disagree" to "7 = very strongly agree. The range of possible scores is 12–84, with higher scores representing higher levels of perceived social support. It is a self-report scale measuring the perceived level of support from family (Items 3, 4, 8, and 11), friends (Items 6, 7, 9, and 12), and significant others (Items 1, 2, 5, and 10) [35].

The fifth part has 40 items on a Health Literacy Measure for Adolescents for measurement of the health literacy of adolescents to evaluate different levels health self-efficacy, access, reading, understanding, appraisal, use, and communication among adolescents. The instrument was adapted from a health literacy measure for adolescents developed and validated by Ghanbari et al. [33]. It uses a 5-point Likert-type scale for its measurements, with ratings from "1 = never" to "5 = always”.

The range of possible scores was 40–200, with higher scores representing higher levels of health literacy. It is a self-report scale that measures health literacy using self-efficacy (items 1–4), access (items 5–9), reading (items 10–14), understanding (items 15–24), appraisal (items 25–29), use (items 30–33) and communication (items 34–40) [33]. The overall standardised Cronbach’s alpha for reliability score of outcome measurement was 0.94 and which was above 0.80 for all independent variables. Data collection was facilitated by three BSc nurses and supervised by one MSc nurse. During school time, classroom teachers facilitated distributing the research questionnaires to a selected group of students.

### Data quality control

Before data collection, orientation was given to the data facilitators for one day on the techniques of data collection, the purpose of data collection, the content of the questionnaires, how to approach the respondents, and how to deal with difficulties that may arise during the data collection period. A pre-test was conducted at Serbo secondary school by taking 5% (32 adolescent students) of the total sample size one week before the actual data collection.

Based on the pre-test results appropriate corrections were made, such as logical the order of some questions in sociodemographic characteristics questions and contextualization was made according the WHO definition of health literacy. An ongoing check-up was performed each day for completeness of the data by the principal investigator during data collection to ensure the quality of the data by checking filled-out questionnaires.

### Data processing and analysis

Following data collection, the data were rechecked for completeness, entered into Epidata version 4.6, and then exported to SPSS version 26.0. Appropriate coding was performed at each step for all variables as necessary. The analysis included descriptive data such as frequencies, percentages, means, and standard deviations. A bivariable logistic regression analysis was performed to sort candidate variables for multivariable logistic regression with a p-value less than 0.25.

A multivariable logistic regression analysis was conducted to identify factors strongly associated with the level of adolescent high school students’ health literacy. Finally, a p-value of less than 0.05 was used to declare the association and an adjusted odds ratio (AOR) at a 95% confidence interval. Multicollinearity was checked to determine the linear correlation between the independent variables using the variance inflation factor (VIF) and tolerance.

None of the variables yielded a variance inflation factor of >10, tolerance of < 0.1. Hosmer and Lemeshow’s test was found to be insignificant (p-value = 0.147), and the Omnibus test was significant (p-value = 0.000), which indicated that the model was fit.

### Ethical considerations

Ethical clearance was obtained from the Institutional Review Board of the Institute of Health of Jimma University [JUIH/IRB/395/23]. The ethical approval letter was submitted to the Jimma town educational office and to all the selected public high schools in Jimma town. Permission was obtained from the educational office and the selected high school governing bodies. Written informed consent was obtained from students whose ages were 18 years and older and from parents or guardians for those less than 18 years.

Following permission from the school directors, students in the 18–19 age group received a consent form and agreed. The students who were in the <18 age group took an information sheet to their parents or guardians and were told to return after the parent or guardian completed the consent form prepared in the local languages (Amharic or Afaan Oromo) days before data collection. Each participant received a detailed information sheet, which included the fact that participation is voluntary and that they have the right to withdraw at any time if they so desire, and which they signed to indicate their agreement to participate. The study only included students who signed consent or assent papers and provided parental or guardian consent.

## Results

### Socio—Demographic characteristics of the study participants

The analysis included 604 (95.26%) participants from 634. Three hundred seventeen (52.5%) of them were male. Three hundred twenty four (53.6%) of them were in 14 to 17 age group. Based on ethnicity, 447 (74.0%) of the participants were Oromo, followed by Amhara 69 (11.4%). Five hundred seventy (94.4%) of them were single. Five hundred eighty six (97%) of the participants were urban residents. Three hundred thirty nine (56.1%) of them were grade 9–10 students. Three hundred twenty four (53.6%) were from employed fathers (Table 1).

**Table 1:** Socio demographic characteristics of adolescent‘s participated in the study in Jimma town Public high school students, Jimma, Oromia, South west Ethiopia, 2023 (n = 604).

Variables	Categories	Frequency	Percent
Age	14–17	324	53.6
	18–19	280	46.4
Gender	Male	317	52.5
	Female	287	47.5
Grade level	9–10	339	56.1
	11–12	265	43.9
Marital status	Single	570	94.4
	Married	34	5.6
Religion	Orthodox	179	29.6
	Muslim	304	50.3
	Protestant	80	13.3
	Catholic	16	2.6
	Others@	25	4.2
Ethnicity	Oromo	447	74.0
	Amhara	69	11.4
	Gurage	28	4.6
	Dawuro	19	3.2
	Silte	25	4.2
	Others$	16	2.6
Area of residence	Urban	586	97.0
	Rural	18	3.0
Father’s job	Employed	324	53.6
	Unemployed	280	46.4
Mother’s job	Employed	231	38.2
	Unemployed	373	61.8
Father’s educational level	Not able to read and write	35	5.8
	Read and write	52	8.6
	Primary (1–8)	143	23.7
	Secondary (9–12)	114	18.9
	Tertiary (above 12)	260	43.0
Mother’s educational level	Not able to read and write	34	5.6
	Read and write	91	15.1
	Primary (1–8)	125	20.5
	Secondary (9–12)	139	23.2
	Tertiary (above 12)	215	35.6

**NB**:

^$^ = Tigre, Kafa, Yem;

^@^ = Wakefata, Hawariyat, Adventist

### Sources of health information for high school adolescent students

**Fig 2:**
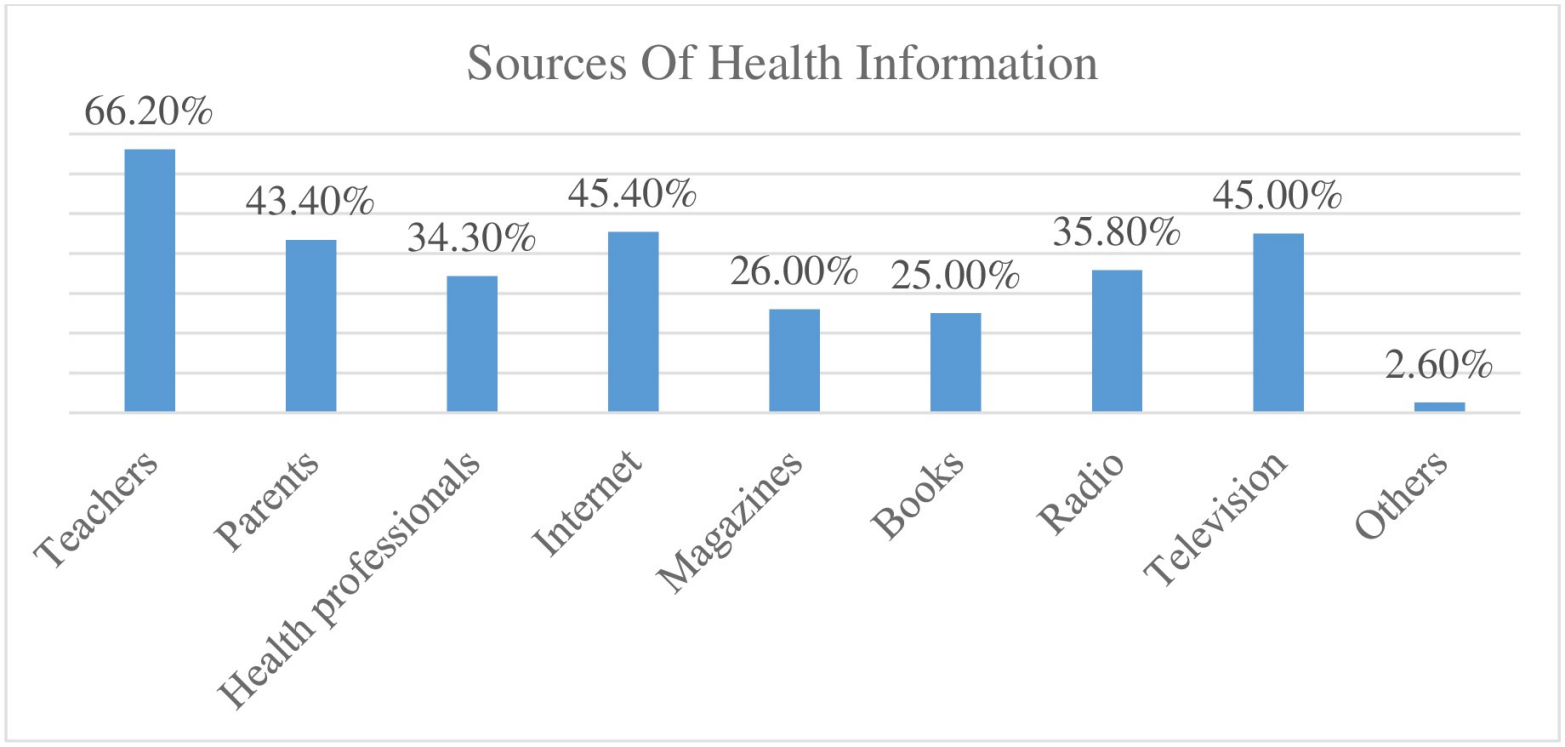
Sources of health information to study level of health literacy and associated factors among Jimma town public high school adolescent students, Jimma, Oromia, South western Ethiopia (n = 604). **NB:** Others = friends, classmates, religious leaders, leaflets. NB: * = Multiple response analysis was computed.

Two in three high school adolescents 400 (66.20%) gained health information from their teachers followed by television 272 (45.00%) (Fig 2).

### School related factors

Nearly three-fourths of the students, 448 (74.2%), reported that their academic performance was medium. Many of them 506 (83.8%) also reported that they had ever attended health-topic education. Most 502 (83.1%) of students, reported that they have interest in health-topic education. Similarly, 442 (73.2%) participants reported that they had ever participated in school clubs (Table 2).

**Table 2:** School related factors to study level of health literacy and associated factors among Jimma town public high school adolescent students, Jimma, Oromia, South western Ethiopia, 2023 (n = 604).

Variables	Categories	Frequency	Percent
Academic performance	High	136	22.5
	Medium	448	74.2
	Low	20	3.3
	Total	604	100.0
Ever attended school classes on health topics	No	98	16.2
	Yes	506	83.8
	Total	604	100.0
Interest in health topics	No	102	16.9
	Yes	502	83.1
	Total	604	100.0
Ever participated in school clubs	No	162	26.8
	Yes	442	73.2
	Total	604	100.0

Regarding school environment scale, 105 (17.4%) of students reported that they strongly agreed that students had lots of chances to help decide things like class activities and rules. Only 174 (28.8%) of them reported that they agree that teachers asked them to work on special classroom projects. Hundred-seventy six students (29.1%) reported that they had a lot of chances to be part in class discussions or activities (Table 3).

**Table 3:** Learning environment scale to study level of health literacy and associated factors among Jimma town public high school adolescent students, Jimma, Oromia, South western Ethiopia, 2023 (n = 604).

Items	Frequency (%)			
	Strongly disagree	Disagree	Agree	Strongly agree
In my school, students have lots of chances to help decide things like class activities and rules	173(28.6)	144(23.8)	182(30.1)	105(17.4)
There are lots of chances for students in my school to talk with a teacher one-on-one	166(27.5)	155(25.7)	180(29.8)	103(17.1)
Teachers ask me to work on special classroom projects	157(26.0)	162(26.8)	174(28.8)	111(18.4)
There are lots of chances for students in my school to get involved in sports, clubs, and other school activities outside of class	177(29.3)	161(26.7)	189(31.3)	77(12.7)
I have a lot of chances to be part of class discussions or activities	164(27.2)	145(24)	176(29.1)	119(19.7)
My teachers notice when I am doing a good job and let me know about it	165(27.3)	149(24.7)	180(29.8)	110(18.2)
The school lets my parents know when I have done something well	158(26.2)	153(25.3)	188(31.1)	105(17.4)
I feel safe at my school	166(27.5)	131(21.7)	164(27.2)	143(23.7)
My teachers praise me when I work hard in school.	165(27.3)	151(25)	183(30.3)	105(17.4)

### Multidimensional perceived social support scale

More than half of students scored high multidimensional perceived social support scale by family 163 (27%), friends 137 (22.7%), and significant others 99 (16.7%) respectively (Table 4).

**Table 4:** Social support scale to study level of health literacy and associated factors among Jimma town public high school adolescent students, Jimma, Oromia, South western Ethiopia, 2023 (n = 604).

Perceived Social Support Scale	Levels of social support		
	Low	Medium	High
Family	107(17.7%)	334(55.3%)	163(27%)
friends	108(17.9%)	359(59.4%)	137(22.7%)
Significant others	61(10.1%)	444(73.5%)	99(16.7%)

### Level of health literacy

**Fig 3:**
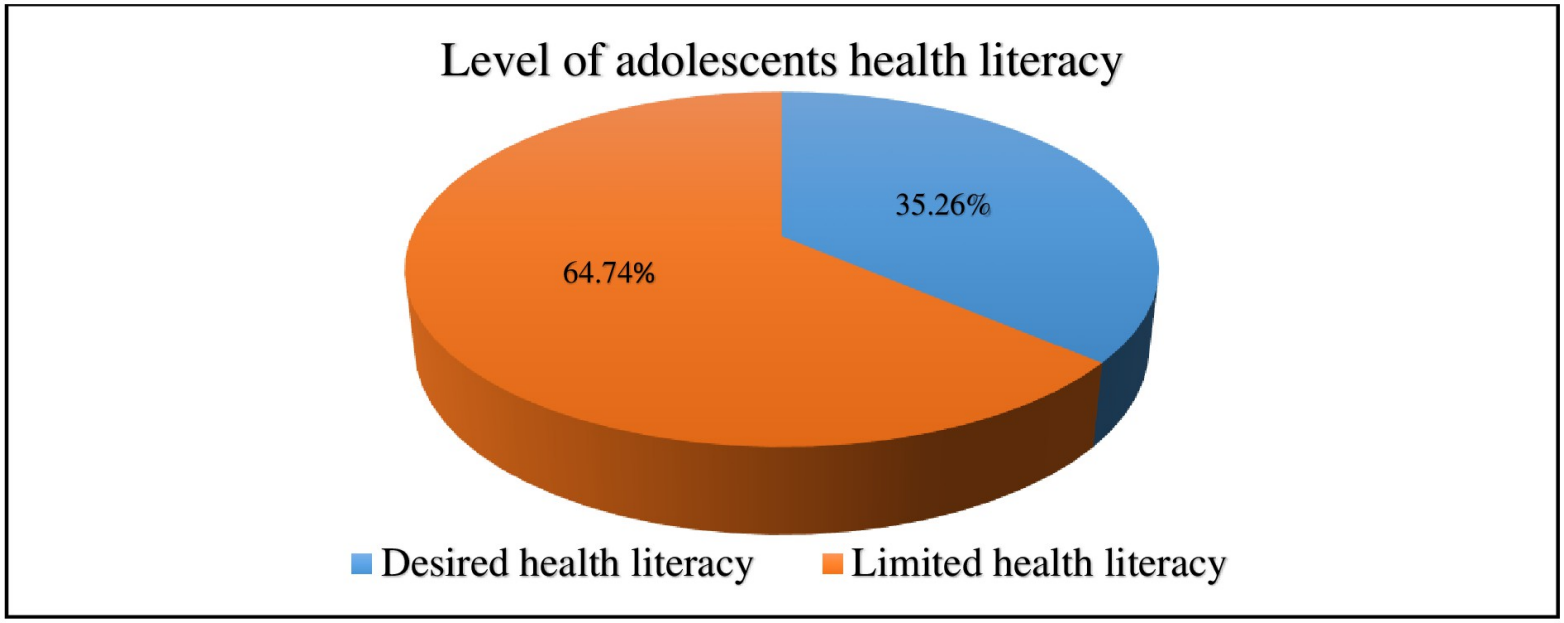
Level of health literacy among Jimma town Public high school adolescent students, Jimma, Oromia, Southwest Ethiopia (n = 604).

Based on the obtained results 213 (35.26%) (95% CI; 31.44, 39.09) had desired health literacy and 391 (64.74%) of participants had limited health literacy, respectively (Fig 3).

### Factors associated with level adolescents’ health literacy

The bivariable logistic regression revealed that 10 variables namely age, grade level, health information from health professionals, fathers’ job, mother’s job, fathers’ education, mother’s education, academic performance, social support from family and social support from friends were identified as candidate variables for multivariable logistic analysis at a p-value less than 0.25. Hosmer and Lemeshow’s test was found to be insignificant (p-value = 0.147) and the Omnibus test was significant (p-value = 0.000) which indicates that the model was fitted.

All candidate variables were entered into a multivariable logistic regression to determine the final factors associated with health literacy. In a multivariable logistic regression 4 variables were found to be the statistically significant associated factors with level of adolescent health literacy at a p-value of < 0.05 at 95% CI. Accordingly, age, grade level, father’s job and mother’s job were associated with adolescent health literacy.

Adolescents in the age of 18 to 19 years had 3.99 times higher odds of health literacy than those whose age was in 14 to 17 years [AOR = 3.99(2.41, 6.60)]. The odds of having health literacy among students in grades 11 and 12 were 2.38 folds higher compared to students in grades 9 and 10 [AOR = 2.38(1.44, 3.95)]. Students whose fathers were employed had 4.20 times higher odds of health literacy than those whose fathers were unemployed [AOR = 4.20(1.98, 8.92)].

Students whose mothers were employed had 4.54 folds higher odds of desired health literacy as compared to those whose mothers were unemployed [AOR = 4.54(2.82, 7.31)] (Table 5).

**Table 5:** Bivariable and multivariable logistic regressions showing factors affecting level of health literacy among Jimma town public high school adolescent Students, Jimma, Oromia, Southwest Ethiopia, 2023 (n = 604).

Variables	Categories	HL Category		COR(95%CI)	AOR(95%CI)	P-values
		Desired	Limited			
Age	14–17	61	263	1	1	
	18–19	152	128	5.12(3.56,7.372)*	3.99(2.41,6.60) **	<0.001
Grade level	9–10	77	262	1	1	
	11–12	136	129	3.59(2.53, 5.09)*	2.38(1.44,3.95)**	0.001
Health professionals	Yes	132	75	1	1	
	No	81	316	0.15(0.10,0.21)*	0.86(0.30,2.53)	0.789
Fathers job	Unemployed	27	253	1	1	
	Employed	186	138	12.63(8.02,19.88)*	4.20(1.98,8.92)**	<0.001
Mother’s job	Unemployed	63	310	1	1	
	Employed	150	81	9.11(6.22,13.36)*	4.54(2.82,7.31)**	<0.001
Father’s Education	No formal education	13	74	1	1	
	Primary	13	130	0.57(0.25,1.29)*	0.47(0.19,1.19)	0.111
	Secondary	32	82	2.22(1.08,4.55)*	0.72(0.29,1.81)	0.484
	Tertiary	155	105	8.40(4.43,15.93)*	1.40(0.56,3.48)	0.475
Mother’s educational level	No formal education	12	113	1	1	
	Primary	21	104	1.90(0.89,4.06)*	1.42(0.58,3.48)	0.446
	Secondary	66	73	8.51(4.31,16.84)*	2.21(0.94,5.20)	0.071
	Tertiary	114	101	10.63(5.53,20.41)*	2.42(1.06,5.50)	0.036
Academic performance	High	59	77	1		
	Medium/Low	154	314	0.64(0.43,0.95)*	0.77(0.45,1.32)	0.344
Social support from family	Low	33	74	1	1	
	Medium	113	221	1.15(0.72,1.83)	1.49(0.80,2.79)	0.214
	High	67	96	1.57(0.94,2.62)	1.38(0.69,2.76)	0.359
Social support from friends	Low	29	79	1	1	
	Medium	125	234	1.46(0.90,2.35)	1.06(0.48,2.35)	0.883
	High	59	78	2.06(1.20,3.55)	1.29(0.46,3.64)	0.629

NB:

* = (P<0.25) in bivariable, 1 = Reference group,

** = statistically significant in multivariable

## Discussion

Health literacy is most commonly defined as the ability of individuals to access, understand, evaluate, and utilize information and services in ways that advance and maintain well-being for themselves and others. This study aimed to examine the level of health literacy and associated factors among adolescents in Jimma town public high schools in South-West Ethiopia. The level of desired health literacy in the present study was (35.26%) (95% CI; 31.44, 39.09). This indicates that a significant number of high school adolescents in Jimma town had no capacity to find, analyse, and comprehend the fundamental health information and services required to make informed health decisions. In the final multivariable logistic regression analysis, age (18–19), being in the 11–12 grade level, having a currently employed father, and having a currently employed mother were found to be independently associated with a higher score of health literacy. The overall prevalence of desired health literacy in the current study (35.26%) is in line with previous research findings done in United Arab Emirates (34.1%) [52] and Nigeria (37.7%) [53].

However, the finding of this study (35.26%) was lower than the findings of previous studies done in China [54], Turkey [55], Indonesia [56], Iran [57], and Malaysia [58] which had reported 48.1%, 43.9%, 64.24%, 62.9%, and 42.1%, respectively. On the contrary, the prevalence of desired health literacy in a study done in the Iran among school adolescents was 28.5% [59], whereas, it was 28.1% and 22% in Guatemala [60] and Ghana [61] respectively.

This difference might be due to socio-economic differences between our study participants and participants in China. Low socioeconomic status in low- and middle-income countries like Ethiopia may hinder access to healthcare and health education, limiting the need for improved healthcare infrastructure [62].

Furthermore, the difference might be due to the variation in sources of participants’ health information. The sources of health information of many adolescents in our study participants from health professionals and the internet were less when compared to sources of information of adolescents in Turkey, which might have decreased their health literacy, making it lower in our study participants [63].

Moreover, school-type differences in the different study settings might have also contributed to the variation. All of the participants in our study were from public high schools, whereas most of the study participants in Indonesia were from private high schools with greater financial resources, allowing them to invest in comprehensive health education programs and resources that contribute to adolescents’ health literacySampling technique and sample size could also be factors in health literacy differences. While we used random sampling techniques and a 634 sample size, the study in Indonesia was done by using convenience sampling techniques with a high sample size (1066).

There were differences in the school type of our study area schools, which were academic public high schools in our study, whereas Indonesian schools included academic, vocational, and Islamic high schools. Moreover, cultural differences in the different study settings might have also contributed to the variation. Adolescents from diverse cultural backgrounds, like in Ethiopia, face challenges in accessing health information due to language barriers, cultural beliefs, and social stigma.

Another reason for these difference might be place of residence, age, field of study, interest in health-related education and sample size affecting their level of health literacy [58].

Participants in our study had greater interest in health-related topics that drive them to actively seek information, engage in reading materials, explore online resources, participate in health-related discussions, and enhance their health literacy. In contrast to a study conducted in Iran, where only 15% of participants expressed a strong interest in health-related issues, over 50% of our participants did [59].

Our study used a larger sample size than a study conducted in Guatemala, which was 210 from 10 schools [60]. Our study participants were students who are considered having more health literacy, where the study in Ghana included street adolescents who lack access to proper health information, have poor educational levels, and have extremely low socioeconomic positions, which constituted the study’s target group in Ghana, in which they find it difficult to successfully acquire and use health information. Our research finding showed that age was positively associated with adolescent health literacy. This finding is consistent with those of studies done in America, China, Japan, Nepal, Bosnia-Herzegovina, and South Africa [38, 42, 64–67]. Age affected adolescents’ health literacy. Younger adolescents may lack knowledge and experience in interpreting health information and many rely on parents or caretakers. However, older adolescents have more autonomy and freedom to manage their health [68].

The results of our study also showed that grade level was strongly associated with adolescent health literacy. This is consistent with previous studies in the United States, China, Japan, and Ghana [38, 64, 65, 69]. This might be due to the amount of health related education and information, which in turn increases health literacy [70]. Adolescents gain more health information and better decision-making skills as they progress through grades. This is due to increased exposure to health information, improved interpretation, and better health-related decision-making. Adolescents learn about various health subjects, develop evaluation skills, and recognise trustworthy sources. Higher grades have better health knowledge, enabling them to make informed decisions that benefit them [71].

Having employed fathers and mothers was also one of the factors that influenced adolescent health literacy. This finding is in line with the study conducted in Iran [44]. Working fathers and mothers significantly improve their health literacy since they have a separate income and may use it to invest in their children to overcome a number of health challenges in a range of areas, notably the utilisation area, in addition to assisting in solving adolescents’ health issues.

### Strengths and limitations of the study

The major strength of this study is that it is the first of its type to assess the level of adolescents’ health literacy and associated factors in Ethiopia. Aside from this, the strength observed in this study was the maximum effort exerted to ensure a random selection of study participants. Thus, generalization to adolescent students in the study area is possible.

The cross‑sectional nature of this study did not allow cause and effect relationship between health literacy and other variables. The study was performed using data from high school adolescent students in Jimma town only, which may affect the generalizability of the results.

It is essential that nurses as one of the largest healthcare professionals should recognize the barriers and facilitators of health literacy in adolescents to effectively measure and build health literacy capacity.

## Supporting information

S1 File (SAV)
